# Association of metformin with lower atrial fibrillation risk among patients with type 2 diabetes mellitus: a population-based dynamic cohort and in vitro studies

**DOI:** 10.1186/s12933-014-0123-x

**Published:** 2014-08-10

**Authors:** Shang-Hung Chang, Lung-Sheng Wu, Meng-Jiun Chiou, Jia-Rou Liu, Kuang-Hui Yu, Chang-Fu Kuo, Ming-Shien Wen, Wei-Jan Chen, Yung-Hsin Yeh, Lai-Chu See

**Affiliations:** Chang Gung University and Department of Cardiology, Chang Gung Memorial Hospital, Kweishan, Taoyuan 333 Taiwan; Department of Public Health, College of Medicine, Chang Gung University, 259 Wen-Hwa 1st Road, Kweishan, Taoyuan 333 Taiwan; Department of Rheumatology, Allergy and Immunology, Chang Gung Memorial Hospital, Kweishan, 333 Taiwan; Biostatistics Core Laboratory, Molecular Medicine Research Center, Chang Gung University, Kweishan, 333 Taiwan

**Keywords:** Metformin, Atrial fibrillation, Myolysis, Oxidative stress, Diabetes mellitus

## Abstract

**Background:**

Atrial fibrillation (AF), an inflammatory process involving arrhythmia, is associated with severe morbidity and mortality and commonly seen in patients with diabetes mellitus (DM). The effect of metformin, the most commonly used medication for patients with DM, on AF has not been investigated. The primary aim of this study was to examine whether metformin prevented the occurrence of AF in type 2 DM patients by analyzing a nationwide, population-based dynamic cohort. Additionally, we investigated the effect of metformin on tachycardia-induced myolysis and oxidative stress in atrial cells.

**Methods:**

The study population included 645,710 patients with type 2 diabetes and not using other anti-diabetic medication from a subset of the Taiwan National Health Insurance Research Database. Of these patients, those who used metformin were categorized as the user group, and the remaining were classified as the non-user group. The time-dependent Cox’s proportional hazard model was used to examine the effect of metformin on AF and the status of metformin use was treated as a time-dependent covariate. HL-1 atrial cells were paced with or without metformin, and then troponin and heavy-chain-myosin were measured as markers of myolysis.

**Results:**

After 13 years of follow-up, 9,983 patients developed AF with an incidence rate of 1.5% (287 per 100,000 person-years). After adjusting for co-morbidities and medications, metformin independently protected the diabetic patients from new-onset AF with a hazard ratio of .81 (95% confidence interval 0.76-0.86, p < 0.0001). Metformin significantly decreased the extent of pacing-induced myolysis and the production of reactive oxygen species.

**Conclusion:**

Metformin use was associated with a decreased risk of AF in patients with type 2 DM who were not using other anti-diabetic medication, probably via attenuation of atrial cell tachycardia-induced myolysis and oxidative stress.

**Electronic supplementary material:**

The online version of this article (doi:10.1186/s12933-014-0123-x) contains supplementary material, which is available to authorized users.

## Introduction

Atrial fibrillation (AF) is the most common tachyarrhythmia and is associated with severe morbidity and mortality in clinical practice. The mechanisms underlying AF are extremely complex, and the current strategy for treating and preventing AF is suboptimal [1,2]. Risk factors for AF include old age, male gender, hypertension, valvular disease, congestive heart failure, and diabetes mellitus (DM). Increasingly, evidence has suggested that inflammation is involved with the pathogenesis of AF associated with DM [3–6]. Metformin, the most commonly used first-line anti-diabetic agent, has been shown to attenuate inflammatory responses and oxidative stress in diabetic patients independent of the anti-hyperglycemic effect [7–14]. AF has been shown to increase oxidative stress and induce structural remodeling in atrial myocytes, including degradation of myofibrils and glycogen deposition (also known as myolysis) [15].

We hypothesized that metformin might provide protection against AF. By analyzing data obtained from a representative dynamic cohort of patients with diabetes in Taiwan, the present study aimed to determine whether or not metformin prevented new onset AF. Two of the advantages of a dynamic cohort (where patients can be recruited at different times) are that the number of participants does not decline over time, and that the aging of study participants over time does not weaken the study. The database used in this study contained a large sample size and provided an excellent opportunity to study the association between metformin use and the occurrence of AF. Additionally, we investigated 1) the effect of metformin on the inhibiting of the generation of reactive oxygen species (ROS), and 2) the attenuation of myolysis induced by tachypacing in atrial cells.

## Methods

### Database

This study used the “Longitudinal Cohort of Diabetes Patients Database (LHDB) 1999-2010,” which is released by the Taiwan National Health Research Institutes for research purposes. The National Health Insurance program, the universal health care system in Taiwan, was launched in 1995 and currently covers 23.72 million enrollees, representing about 99% of the national population. In the LHDB, a first diagnosis of DM between 1999 and 2010 is defined as: (1) the patient had at least one hospitalization with DM as one of the diagnoses (ICD-9-CM code 250 after 2000, or Acode A181 before 2000) or the patient used DM medication (Additional file 1: Supplement 1); or (2) the patient had at least two outpatient visits due to DM in the same year; and (3) the patient had not been hospitalized or treated as an outpatient due to DM before 1999. Approximately 120,000 eligible patients were randomly selected for each year from 1999 to 2010, and their original claims data including admission, outpatient, and medication use were stored in LHDB. For the eligible DM patients, the LHDB includes: (1) data after the DM diagnosis, (2) data before the diagnosis, and (3) data on the treatments for diseases other than DM. Rigid criteria were ensured diagnostic validity and minimized potential misclassification. The LHDB has been tested and confirmed by many other studies to be representative of the Taiwanese DM population [16–19]. The identification number of each patient was encrypted as part of an established privacy policy, and therefore informed consent was not required. This study was approved by the Institutional Review Board of the Chang Gung Medical Foundation, Taiwan.

### Study cohorts

**Figure 1:**
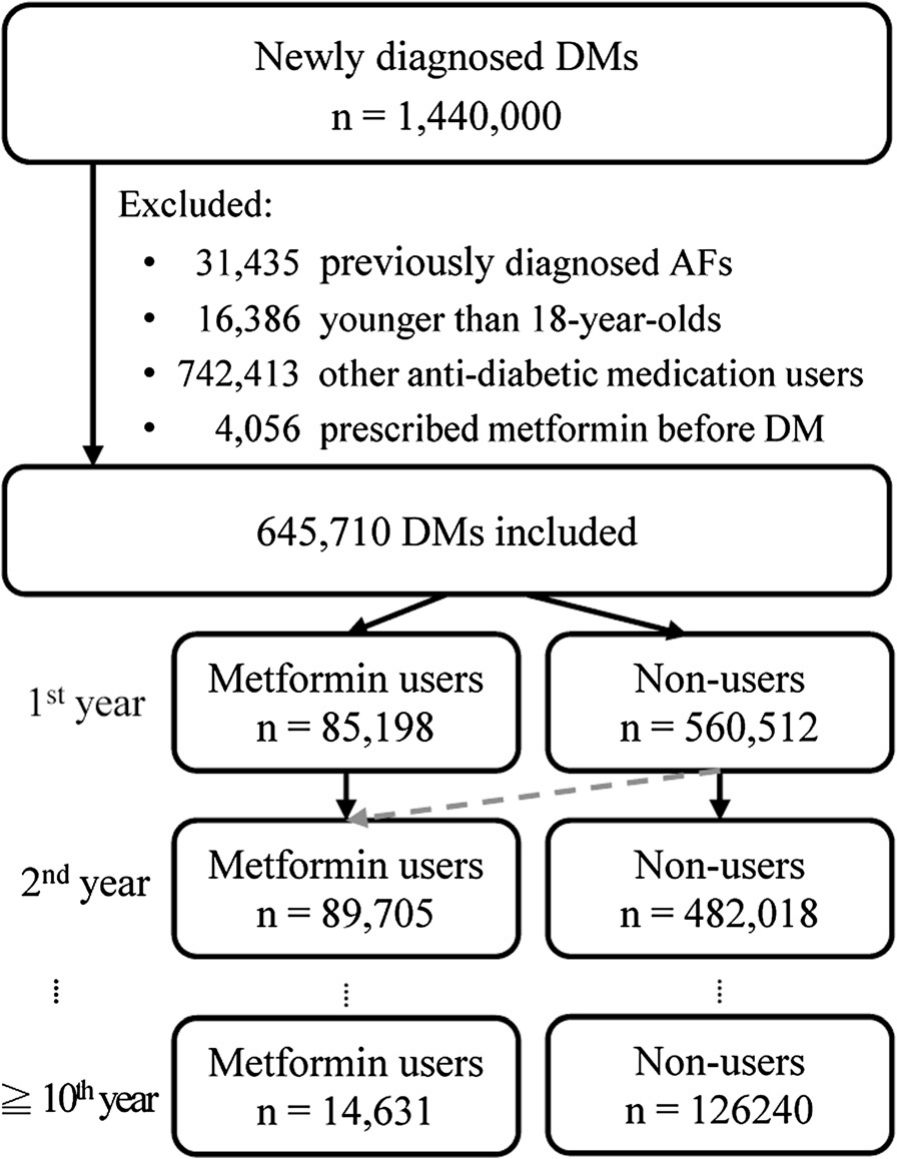
**Flowchart of subject selection.**

The patients who were diagnosed with AF (ICD-9-CM code 427.31) prior to DM, who had two prescriptions for insulin or any anti-diabetic medication other than metformin, or who were younger than 18 years were excluded. The status of metformin use could be changed over time to reflect the actual prescription (Figure 1). The primary outcome was defined as new onset AF during the follow-up period (ICD-9-CM code 427.31, coded two consecutive times). The medications are listed in Additional file 1: Supplement 1, and the ICD-9-CM codes of co-morbidities are listed in Additional file 1: Supplement 2.

### Statistical analysis

The crude incidence rate of AF was calculated as the total number of AF events during the follow-up period divided by person-years at-risk. The person-years at-risk was defined as the sum of patients from the diagnosis of DM to the diagnosis of first AF event, dropout from the national health insurance program, death, or December 31, 2011, whichever came first. The chi-square test or independent *t*-test was used to compare data between the two study groups in univariate analysis. Chronic diseases (hypertension, congestive heart failure, chronic kidney disease, asthma, hyperthyroidism, myocardial infarction, ischemic stroke, sleep apnea syndrome, and peripheral arterial disease) and medication (metformin, anti-hypertensives, and statins) were time dependent. Considering the nature of chronic diseases and consistent prescriptions of medication to reduce blood sugar, blood pressure, or blood lipid, these time-dependent covariates were coded as zero before diagnosis or prescription, and were coded as one after being diagnosed or being prescribed. Hazard ratios (HRs) and their 95% confidence intervals (CIs) were computed. The significance level of this study was 0.05.

### Cell culture and tachypacing

Most chemicals were purchased from Sigma (St Louis, MO, USA). HL-1 atrial myocytes were maintained in Claycomb medium with or without 1 mM of metformin (Sigma) for 2 hours. The treated atrial myocytes were then subjected to field stimulation as described previously [15,20]. To induce tachycardia, HL-1 cells (≥1 × 10^6 cells) were cultured on 4-well rectangular dishes (Nuclon, Netherlands) and placed into C-Dish 100TM-Culture Dishes (IonOptix, Milton, MA, USA). The cells were paced with 10-ms stimuli of 40-V intensity at 4 Hz. Capture efficiency > 90% was confirmed by microscopic examination and by shortening of the action potential duration as described previously [15].

### Detection of intracellular oxidative stress and immunohistochemical and cytochemical analyses

Intracellular oxidative stress from ROS was measured with fluorescent dye DCF-DA (2,7-dichlorofluorescein diacetate) and detected by confocal microscopy (Leica TCS SP2, Carl Zeiss, Jena, Germany) after pacing. DCF-DA was excited at 488 nm with an argon laser, and emission at 525 nm was recorded. Two-dimensional images (512 × 512 pixels) were acquired. Immunohistochemical and cytochemical analyses were performed by confocal microscopy using myosin heavy chain (MHC, Abcam) primary antibodies followed by fluorescein isothiocyanate (FITC) conjugated secondary antibodies (Chemicon, Temecula, CA, USA). Nuclei were visualized by diamidino-2-phenylindole (DAPI)-staining. Myosin degradation was quantified as the cytoplasmic MHC-area divided by the nuclear area. The relative expression levels of MHC were normalized to the control level. For each analysis, at least five random fields were chosen to observe more than 30 myocytes.

### Immunoblotting

Equal amounts of protein in sodium dodecyl sulfate-polyacrylamide gel (SDS-PAGE) sample buffer was sonicated and subjected to electrophoresis on 12.5% SDS-PAGE. After the sample had been transferred to nitrocellulose membranes (Stratagene, Netherlands), the membranes were incubated with primary antibodies against cTroponin I (Fitzgerald Industries) or tubulin (Santa Cruz, Delaware Avenue, CA, USA). Signals were detected by an enhanced chemiluminescence substrate-detection method (Amersham, Netherlands) and quantified by densitometry. Signal bands were in the linear immunoreactive range, and they were expressed relative to tubulin.

## Results

### Cohort study

From 1999 to 2010, 645,710 patients who were newly diagnosed type 2 DM, were not using insulin, were older than 18 years, and had not used other oral anti-diabetics were enrolled in this study. The mean follow-up duration was 5.4 years. Within the first year of DM diagnosis, 85,198 (13.2%) patients used metformin and 560,512 patients (86.8%) did not. The mean age of the study population was 58.6 ± 17.1 years, and males accounted for 49.8% of the patients. The demographic characteristics, relevant co-morbidities, and medications used in the first and ≥10th year after the diagnosis of DM are shown in Table 1. During the follow-up period after the diagnosis of DM, the metformin group had higher incidences of hypertension and sleep apnea syndrome, while the non-user group had higher incidences of congestive heart failure, chronic kidney disease, asthma, myocardial infarction, stroke, and peripheral arterial disease. In general, anti-hypertensive drugs were commonly used among the patients with DM, and the proportion increased from 62.8% to 70.7% during the follow-up period. Statins were the second most commonly used drug, with an increase in prevalence from 20.5% to 27.8%. The metformin group took more anti-hypertensives and statins than the non-user group (Additional file 1: Supplement 3).

**Table 1:** **Demographic and comorbidity characteristics of the DM patients diagnosed by status of metformin use**

	**First year (n = 645,710)**			**≥10th year (n = 140,871)**		
	**Non-users** ^*^	**Users** ^**†**^	**P**	**Non-users** ^*^	**Users** ^**†**^	**P**
	**(n = 560,512)**	**(n = 85,198)**		**(n = 126,240)**	**(n = 14,631)**	
Sex			<.0001^‡^			0.2560^‡^
Female	281325 (50.2%)	44179 (51.9%)		73268 (58.0%)	8420 (57.6%)	
Male	279187 (49.8%)	41019 (48.2%)		52972 (42.0%)	6211 (42.5%)	
Age, years						
Mean ± SD	58.6 ± 17.1	57.0 ± 14.8	<.0001^§^	53.7 ± 17.0	55.4 ± 15.0	<.0001^§^
Co-morbidity						
Hypertension	301047 (53.7%)	54032 (63.4%)	<.0001^‡^	71480 (56.6%)	10601 (72.5%)	<.0001^‡^
Congestive heart failure	42886 (7.7%)	5162 (6.1%)	<.0001^‡^	11056 (8.8%)	1507 (10.3%)	<.0001^‡^
Chronic kidney disease	75572 (13.5%)	7337 (8.6%)	<.0001^‡^	22788 (18.1%)	2449 (16.7%)	<.0001^‡^
Asthma	77366 (13.8%)	11888 (14.0%)	0.2352^‡^	22437(17.8%)	2898 (19.8%)	<.0001^‡^
Hyperthyroidism	22517 (4.0%)	3042 (3.6%)	<.0001^‡^	8286 (6.6%)	877 (6.0%)	0.0082^‡^
Myocardial infarction	8542 (1.5%)	1165 (1.4%)	0.0005^‡^	2186 (1.7%)	305 (2.1%)	0.0022^‡^
Ischemic stroke	46557 (8.3%)	6207 (7.3%)	<.0001^‡^	12739 (10.1%)	1818 (12.4%)	<.0001^‡^
Sleep apnea syndrome	3949 (0.7%)	1058 (1.2%)	<.0001^‡^	1874 (1.5%)	319 (2.2%)	<.0001^‡^
Peripheral arterial disease	4238 (0.8%)	399 (0.5%)	<.0001^‡^	1387 (1.1%)	165 (1.1%)	0.7500^‡^
Medication						
Anti-hypertensives^¶^	345487 (61.6%)	60278 (70.8%)	<.0001^‡^	87769 (69.5%)	11896 (81.3%)	<.0001^‡^
Statin	100497 (17.9%)	31918 (37.5%)	<.0001^‡^	32403 (25.7%)	6755 (46.2%)	<.0001^‡^

^*^Nonusers were adults (age≧18 years) with no record of metformin use.

^†^Users were adults who used metformin.

^‡^Chi-squared test.

^§^Independent *t* test.

^¶^Angiotensin receptor blockers, angiotensin converting enzyme inhibitors, beta blockers, and calcium channel blockers.

**Figure 2:**
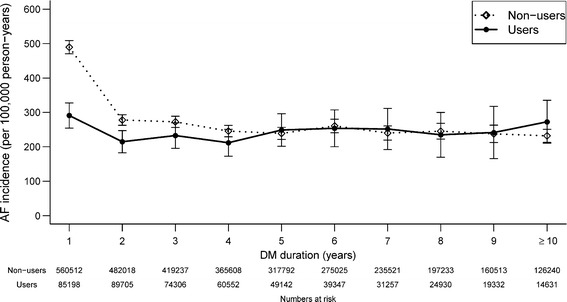
**Atrial fibrillation (AF) incidence (per 100,000 person-years) vs. DM duration by use of metformin among DM patients, Taiwan 1999–2010.**

**Figure 3:**
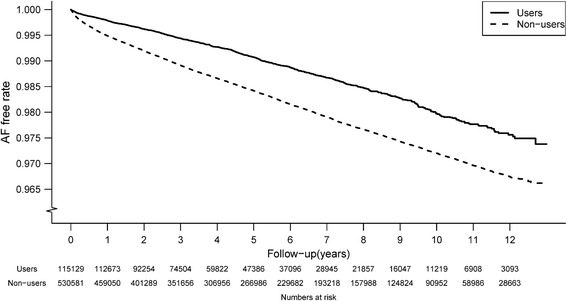
**AF-free survival rate (n = 645,710) for diabetic patients with and without metformin use.** AF = atrial fibrillation. Solid line is for the metformin users and broken line is for the non-users (p < 0.0001).

The overall AF rate was 287 per 100,000 person-years, and the AF rate for the metformin user group was significantly lower than that of the nonuser group (245 for metformin users and 293 for nonusers, p < 0.0001). From the first to the second year after the diagnosis of DM, the metformin group had significantly lower incidence rates of AF than the non-user group (Figure 2). Figure 3 demonstrates Kaplan-Meier curves of the AF-free survival rates among the diabetic patients who did and did not use metformin. The metformin group had a significantly higher cumulative AF-free rate during the 13 years of follow-up than the non-user group (p < 0.001).

Unadjusted and adjusted HRs of AF calculated by Cox multivariate regression analyses are presented in Table 2. After adjusting for age, sex, hypertension, congestive heart failure, chronic kidney disease, asthma, myocardial infarction, ischemic stroke, peripheral arterial disease, the use of anti-hypertensives, and statins, the metformin group had a significantly lower AF occurrence rate with a HR of 0.81 (95% CI 0.76-0.86, p < 0.001).

**Table 2:** **Unadjusted and adjusted hazard ratios (HR) of atrial fibrillation (AF) among patients with diabetes in Taiwan from 1999–2010**

	**Unadjusted HR**	**P**	**Adjusted HR**	**P**
	**(95% CI)**		**(95% CI)**	
Age				
18-49	1.00 (reference)		1.00 (reference)	
50-64	4.49 (4.01-5.03)	<.0001	3.17 (2.83-3.56)	<.0001
≥65	16.64 (14.97-18.49)	<.0001	8.10 (7.25-9.04)	<.0001
Sex				
Female	1.00 (reference)		1.00 (reference)	
Male	1.38 (1.33-1.44)	<.0001	1.33 (1.28-1.38)	<.0001
Comorbidity				
Hypertension	4.39 (4.15-4.63)	<.0001	1.27 (1.19-1.35)	<.0001
Congestive heart failure	5.48 (5.25-5.71)	<.0001	2.72 (2.60-2.84)	<.0001
Chronic kidney disease	1.75 (1.67-1.84)	<.0001	- -	- -
Asthma	1.96 (1.87-2.05)	<.0001	1.10 (1.05-1.15)	<.0001
Hyperthyroidism	1.00 (0.92-1.10)	0.9433		
Myocardial infarction	3.00 (2.73-3.29)	<.0001	1.27 (1.15-1.39)	<.0001
Ischemic stroke	2.27 (2.16-2.39)	<.0001	1.06 (1.01-1.12)	0.0192
Sleep apnea syndrome	0.95 (0.78-1.16)	0.6049		
Peripheral arterial disease	2.52 (2.20-2.90)	<.0001	1.22 (1.06-1.40)	0.0055
Medication				
Metformin	0.80 (0.75-0.85)	<.0001	0.81 (0.76-0.86)	<.0001
Anti-hypertensives^*^	6.87 (6.36-7.42)	<.0001	3.08 (2.83-3.35)	<.0001
Statin	1.18 (1.13-1.23)	<.0001	0.85 (0.81-0.89)	<.0001

^*^angiotensin receptor blockers, angiotensin converting enzyme inhibitors, beta blockers, and calcium channel blockers.

### Metformin attenuated tachycardia-induced oxidative stress and atrial remodeling

**Figure 4:**
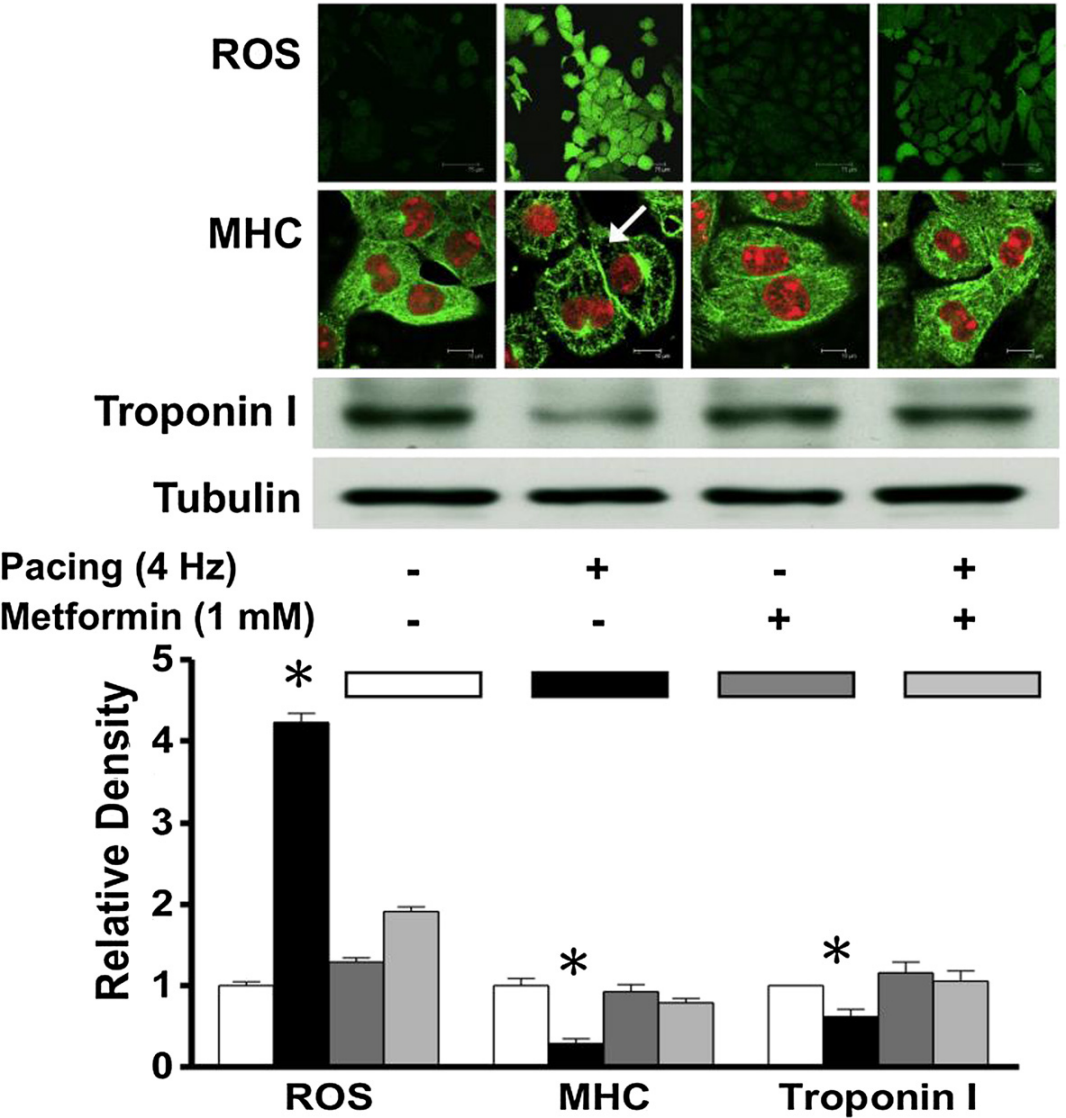
**HL-1 cells were paced at 4Hz for 24 hours.** Figure 4 shows that tachypacing induced intracellular oxidative stress from ROS in atrial myocytes and that metformin reversed this effect. Tachycardia induced myofibril degradation quantified by myosin heavy chain and troponin-I were also rescued by metformin of 1 mM.

A large body of evidence suggests that there is a link between oxidative stress and AF [21]. During tachyarrhythmia, substantial oxidative damage and cellular (ionic and structural) remodeling occurs in atrial myocardium that can induce AF [22]. We used MHC and troponin I degradation in cultured HL-1 atrial myocytes to evaluate the effects of metformin on preventing tachycardia-induced oxidative stress and on reducing the extent of cellular remodeling. HL-1 cells were paced at 4 Hz for 24 hours. Figure 4 shows that tachypacing induced intracellular oxidative stress from reactive oxygen species (ROS) in atrial myocytes, and that metformin reversed this effect. Tachycardia-induced myofibril degradation quantified by MHC and troponin-I was also rescued by 1 mM of metformin.

## Discussion

The main findings of this long-term population-based retrospective cohort study are as follows. (1) Metformin was associated with a decreased risk of new onset AF in type 2 DM patients not using other anti-diabetics; (2) this protective effect remained for at least two years after the use of metformin; (3) metformin inhibited the tachypacing-induced generation of ROS in atrial myocytes; and (4) metformin attenuated tachypacing-induced myofibril degradation. The current study is the first population-based epidemiological investigation on the association between the use of metformin and development of AF. This study is also the first to suggest a protective effect of metformin against tachypacing-induced cellular injury in atrial myocytes.

DM increases the incidence of AF through unknown mechanisms [3–5,23]. Anatomical (increasing atrial size), biochemical (inflammation), and clinical (coronary disease and heart failure) changes have been suggested as possible factors involved in the pathogenesis of AF [24–26]. In the current cohort study, the metformin user and non-user groups had different profiles of co-morbidities and relevant medications, which were adjusted for our analysis. However, anatomical and biochemical characteristics were not available in the dataset.

This study demonstrated that metformin protected the diabetic patients from AF. This protective effect, however, seemed to diminish two to three years after the diagnosis of DM. This weakening of the protective effect of metformin after the third year may be attributable to common and gradual anatomical and biochemical deterioration because the co-morbidities were similar between the metformin user and non-user groups after 13 years of follow-up. The exact reason for the diminishing effect, however, remains to be investigated in future studies.

The studied population might be a subset of “early stage” of DM patients since they either used metformin or non-pharmacological tools to manage their sugar, although biochemical data was not available. This study, however, strongly hints that earlier use of metformin along diet control and physical activities might offer unexpected benefits in preventing arrhythmia. On the other hand, the effect of other anti-diabetics was not been evaluated in this study. For example, thiazolidinediones, another commonly used anti-diabetic that acts by activating peroxisome proliferator-activated receptor gamma, has also been shown to decrease new onset AF in patients with type 2 DM not using insulin [19]. This protection is thought to be through anti-inflammatory and anti-oxidant mechanisms, which have been well documented [27–30]. Metformin has also been shown to have anti-inflammatory and anti-oxidant properties [7–14,31]. Since oxidative stress mediates tachycardia-induced cellular remodeling, the protection offered by metformin may be due to inhibition of inflammation and oxidation [15]. The effect of sulfonylureas, glucosidase inhibitors, dipeptidyl peptidase-4 inhibitors, and glucagon-like peptide 1 receptor agonist were not investigated either, and their connection with AF is completely unknown.

This is a large population-based and long-term observational study. The diagnosis of AF as well as other diseases and the use of medications came from a nationwide registry in Taiwan. Because of the limitations inherent in the design of this study, it is possible that potential registration errors, bias, and confounding factors may have existed to prevent the establishment of a solid relationship between the use of metformin and the incidence of AF. For example, impaired renal function as an ascertainment bias might interfere with the prescription of metformin. Although we included diagnosis of chronic renal disease in the sub-analysis of regression model and yielded similar results (Additional file 1: Supplement 4), the data available do not eliminate this possible confounding factor entirely. A lack of laboratory data such as hemoglobin A1c also prevented further detailed analysis and classification. Nevertheless, our findings are still important in that they provide significant evidence with regard the association between metformin usage and the lower incidence of AF in DM patients in a large population over a long period of time. The findings may help to elucidate the role of metformin and lead to other potential therapeutic targets in clinically reducing the incidence of AF. Further parallel cohort observations or strict randomized studies are warranted to clarify and validate our findings.

The in vitro experiment provides the first piece of evidence that metformin may be able to attenuate tachycardia-induced oxidative stress generation and subsequent atrial myocyte remodeling, which are known to be substantially involved in the complex and multifactorial pathogenesis of AF. The results of this experiment did not directly explain the mechanism by which metformin was able to reduce the incidence of AF in DM patients, but they did suggest that metformin was protective against AF-related adverse remodeling. More comprehensive and conclusive clinical or animal experiments are needed to elucidate why or how metformin offers protection.

## Conclusions and clinical implications

DM is a strong risk factor for AF. While metformin is a safe and cost-effective drug for most diabetic patients before using any other anti-diabetics, our study suggests that metformin might also offer the additional benefits of preventing new AF events. In a clinical setting, more prescriptions of metformin for diabetic patients should be considered beyond sugar control. Metformin is protective against oxidative stress and adverse atrial remodeling during tachycardia. Further animal and clinical studies are warranted to validate our findings and clarify the mechanisms.

## Additional file

Additional file 1:
**Supplement 1.** The drugs analyzed are listed as following. Supplement 2 ICD-9-CM codes for of co-morbidities analyzed in this study Supplement 3. Medication use vs. DM duration by status of metformin use among DM patients, Taiwan 1999–2010. Solid lines indicate metformin users and broken lines indicate metformin nonusers. Blue and red lines indicate anti-hypertensives and statin respectively. Supplement 4 Sub-analysis of chronic kidney disease. AF incidence vs. DM duration by status of metformin use and chronic kidney disease among DM patients, Taiwan 1999–2010. Solid lines indicate metformin users and broken lines indicate metformin nonusers. Blue and black lines indicate patients with and without chronic kidney disease respectively.

## Abbreviations

AF: Atrial fibrillation
DM: Diabetes mellitus
ROS: Reactive oxygen species
LHDB: Longitudinal cohort of diabetes patients database
MHC: Myosin heavy chain
