# Supplementary material for: Association of metformin with lower atrial fibrillation risk among patients with type 2 diabetes mellitus: a population-based dynamic cohort and in vitro studies
Source: Cardiovasc Diabetol. 2014 Aug 10;13:123. doi: 10.1186/s12933-014-0123-x (PMC4149273; doi:10.1186/s12933-014-0123-x)

Additional file

**Supplement 1**

**The drugs analyzed are listed as following.**

**Insulins**:

insulin aspart;

insulin detemir;

insulin glargine;

insulin human;

insulin lispro;

**ARBs and ACEis**:

captopril;

enalapril;

fosinopril;

lisinopril;

losartan;

olmesartan;

perindopril;;

quinapril;

ramipril;

telmisartan

valsartan

**Beta blockers:**

alprenolol;

atenolol;

carvedilol;

labetalol;

metoprolol;

nadolol;

pindolol;

propranolol;

**Calcium channel blockers**:

amlodipine;

diltiazem;

felodipine;

lacidipine;

lercanidipine;

nicardipine;

nifedipine;

verapamil;

**Statins**:

atorvastatin;

fluvastatin;

lovastatin;

pravastatin;

rosuvastatin;

simvastatin

**Thiazolidinediones:**

pioglitazone;

rosiglitazone;

**Sulfonylurea:**

chlorpropamide;

glibenclamide;

glibornuride;

gliclazide;

glimepiride;

glipizide;

gliquidone;

tolazamide

tolbutamide

**Dipeptidyl peptidase-4 inhibitors:**

linagliptin;

sitagliptin;

saxagliptin

vildagliptin

**Glucosidase inhibitors**

acarbose

Supplement 2

ICD-9-CM codes for of co-morbidities analyzed in this study

| Co-morbidities | ICD9 codes |
| --- | --- |
| AF | 427.31 |
| DM | 250 |
| Hypertension | 401-404 |
| Congestive heart failure | 428 |
| Chronic kidney disease | 580-589 |
| Asthma/COPD | 493 |
| Hyperthyroidism | 242 |
| Myocardial infarction | 410, 412 |
| Ischemic stroke | 433, 434 |
| Sleep apnea syndrome | 3272, 78051, 78053, 78057 |
| Peripheral arterial disease | 444 |

Supplement 3. Medication use vs. DM duration by status of metformin use among DM patients, Taiwan 1999-2010. Solid lines indicate metformin users and broken lines indicate metformin nonusers. Blue and red lines indicate anti-hypertensives and statin respectively.


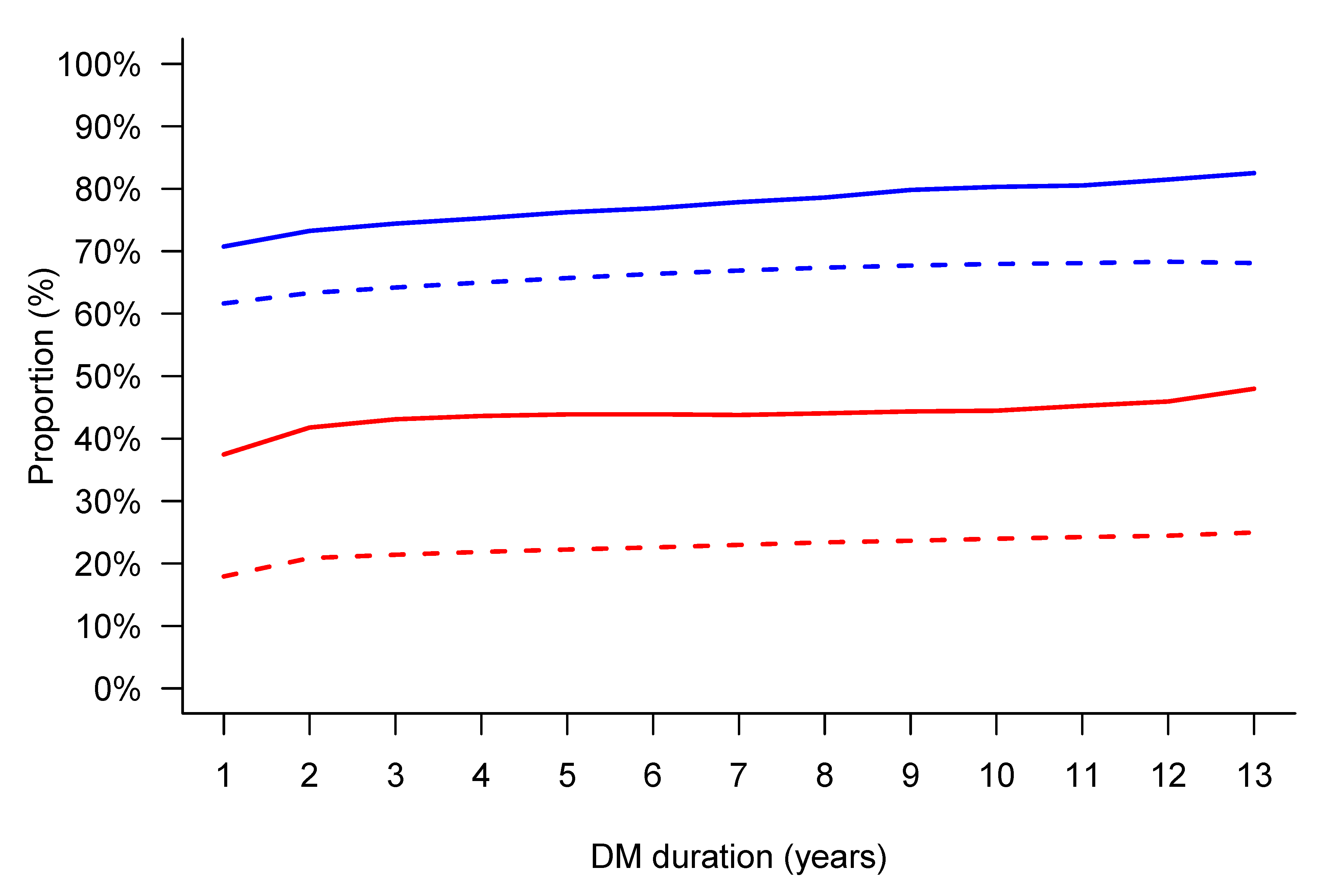


Supplement 4

Sub-analysis of chronic kidney disease. AF incidence vs. DM duration by status of metformin use and chronic kidney disease among DM patients, Taiwan 1999-2010. Solid lines indicate metformin users and broken lines indicate metformin nonusers. Blue and black lines indicate patients with and without chronic kidney disease respectively.


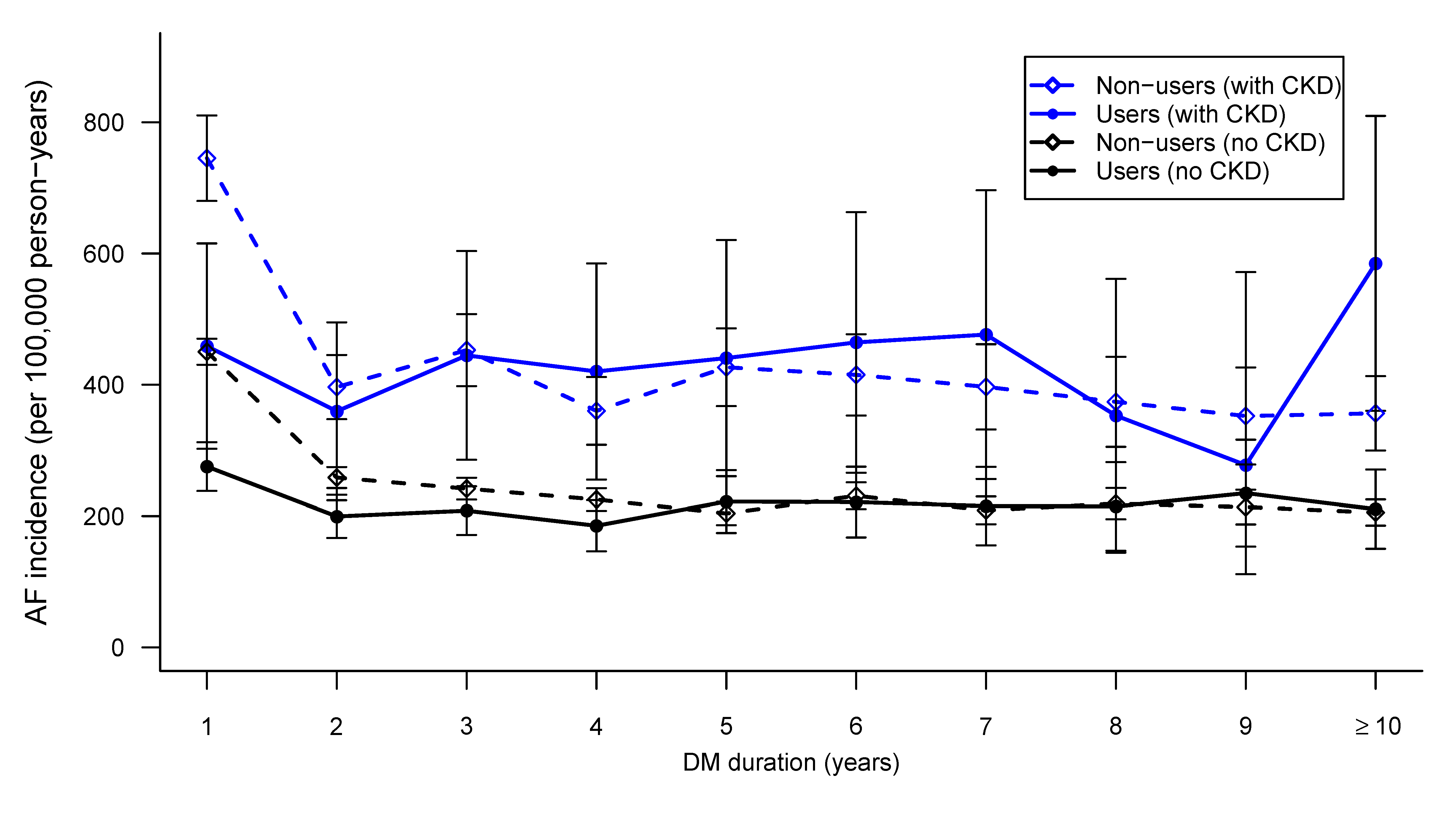

Supplement: Additional file 1: — Supplement 1. The drugs analyzed are listed as following. Supplement 2 ICD-9-CM codes for of co-morbidities analyzed in this study Supplement 3. Medication use vs. DM duration by status of metformin use among DM patients, Taiwan 1999–2010. Solid lines indicate metformin users and broken lines indicate metformin nonusers. Blue and red lines indicate anti-hypertensives and statin respectively. Supplement 4 Sub-analysis of chronic kidney disease. AF incidence vs. DM duration by status of metformin use and chronic kidney disease among DM patients, Taiwan 1999–2010. Solid lines indicate metformin users and broken lines indicate metformin nonusers. Blue and black lines indicate patients with and without chronic kidney disease respectively. [file 12933_2014_123_MOESM1_ESM.doc]
